# New Prospective Phosphodiesterase Inhibitors: Phosphorylated Oxazole Derivatives in Treatment of Hypertension

**DOI:** 10.34172/apb.2023.044

**Published:** 2022-04-04

**Authors:** Iryna V. Nizhenkovska, Kateryna V. Matskevych, Oksana I. Golovchenko, Oleksandr V. Golovchenko, Antonina D. Kustovska, Mikhaeel Van

**Affiliations:** ^1^Bogomolets National Medical University, Kyiv, Ukraine.; ^2^V.P. Kukhar Institute of Bioorganic Chemistry and Petrochemistry of the National Academy of Sciences of Ukraine, Kyiv, Ukraine.; ^3^National Aviation University, Kyiv, Ukraine.

**Keywords:** Hypertension, Molecular docking, Phosphodiesterase, Phosphodiesterase III inhibitors, Phosphorilated oxazole derivatives

## Abstract

***Purpose:*** One of the promising chemical groups for the development of new antihypertensive medicines, the action of which is associated with the inhibition of phosphodiesterase III (PDE3) activity, are phosphorylated oxazole derivatives (OVPs). This study aimed to prove experimentally the presence of the OVPs antihypertensive effect associated with decreasing of PDE activity and to justify its molecular mechanism.

***Methods:*** An experimental study of the effect of OVPs on phosphodiesterase activity was performed on Wistar rats. Determination of PDE activity was performed by fluorimetric method using umbelliferon in blood serum and organs. The docking method was used to investigate the potential molecular mechanisms of the antihypertensive action of OVPs with PDE3.

***Results:*** The introduction of OVP-1 50 mg/kg, as a leader compound, led to the restoration of PDE activity in the aorta, heart and serum of rats with hypertension to the values observed in the intact group. This may indicate the possibility of the development of vasodilating action of OVPs by the influence of the latter on the increase in cGMP synthesis due to inhibition of PDE activity. The calculated results of molecular docking of ligands OVPs to the active site of PDE3 showed that all test compounds have a common type of complexation due to phosphonate groups, piperidine rings, side and terminal phenyl and methylphenyl groups.

***Conclusion:*** The analysis of the obtained results both *in vivo* and *in silico* showed that phosphorylated oxazole derivatives represent a new platform for further studies as phosphodiesterase III inhibitors with antihypertensive activity.

## Introduction

###  Hypertension as a global disease

 According to the WHO in 2021, hypertension is observed in more than a billion people worldwide.^1^ An increase in blood pressure is usually associated with a violation of the factors that regulate the activity of the cardiovascular system.^2^ Typical complications of hypertension are hypertrophy and hyperplasia of cardiovascular smooth muscle, dysregulation of calcium metabolism and changes in hormonal-cell interactions.^3-5^ This leads to such consequences as stroke, heart attack, heart and kidney failure.^6^ Therefore, the current direction of modern pharmacology and pharmacy is the search and study of molecular mechanisms of antihypertensive action of new original compounds. Despite the presence of a large number of antihypertensive drugs at the world pharmaceutical market, the effectiveness of treatment of hypertension does not exceed 20%,^7-9^ due to the significant number of adverse reactions caused by them, addiction to them and the emergence of resistant forms of hypertension. One of the promising chemical groups for the development of new antihypertensive drugs, the action of which is associated with the inhibition of phosphodiesterase III activity, are phosphorylated oxazole derivatives.^10,11^ Since the blockade of phosphodiesterase plays an essential role in the mechanisms of action of many antihypertensive drugs,^12,13^ this fact stimulated us to study the mechanism of antihypertensive action of phosphorylated oxazole derivatives through its effect on the enzyme. The purpose of the study is to prove experimentally the presence of the OVPs antihypertensive effect associated with decreasing of PDE activity and to justify its molecular mechanism.

###  Phosphodiesterase III

**Figure 1:**
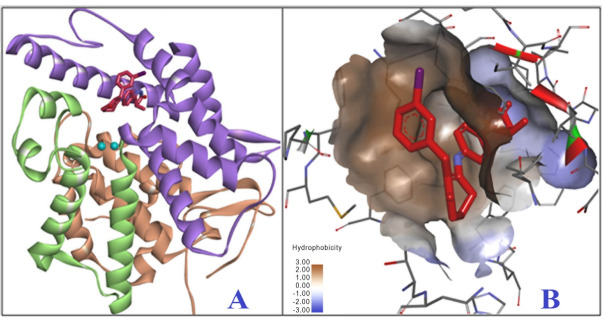
The structure of the catalytic domain of phosphodiesterase 3B. **A** is the general structure of the catalytic domain of PDE3B. **B** is an image of the surface of the active center of PDE3B associated with the MERCK1 inhibitor.

 Of the eleven known families of phosphodiesterases, which differ in their primary and secondary structure, substrate affinity, response to effectors of different nature and mechanism of regulation, phosphodiesterase III (PDE3) has a clinical importance due to its role in the regulation of heart muscle, vascular smooth muscle and platelet aggregation. Moreover, isoforms of PDE3A and PDE3B are expressed in vascular smooth muscle cells and modulate their contraction. The enzyme PDE3 in mammals occurs in two isoforms − PDE3A and PDE3B. These structurally similar isoforms contain an N-terminal domain associated with the localization of the enzyme, a catalytic and a C-terminal domain. The main differences in PDE3A and PDE3B isoforms are the fragment with 44 amino acid residues in the catalytic domain and the N-terminal part.^14^ It should be noted that this fragment is unique to the PDE3 family and is considered a key factor in determining the structure of enzyme inhibitors.^15,16^ The structure of the catalytic domain of PDE3 is presented in Figure 1. The structure of the catalytic domain PDE3B consists of three subdomains (Figure 1A)^17^: N-terminal subdomain (light brown), linker subdomain (light green), C-terminal subdomain (purple). Also, Figure 1A shows the enzyme-linked inhibitor MERCK1 and Mg^2+^ ions (blue) in red. At the boundary of these domains, a deep hydrophobic pocket is formed from amino acid residues specific for PDE3. This hydrophobic pocket is an active site with four subsites (Figure 1B): metal-binding area, main pocket, hydrophobic pocket, lid area.

 According to the literature, PDE3B with affinity for cAMP and cGMP, hydrolyzes them to AMP and GMP with the participation of an invariant glutamine residue, which provides binding of purine cycles of cAMP and cGMP in the active site.^18^ In this case, the activity of PDE3B is regulated in several ways - phosphorylation of two regions (P1 and P2) of the N-terminal subdomain by protein kinases A and B.^16^

###  Known phosphodiesterase III inhibitors

**Figure 2:**
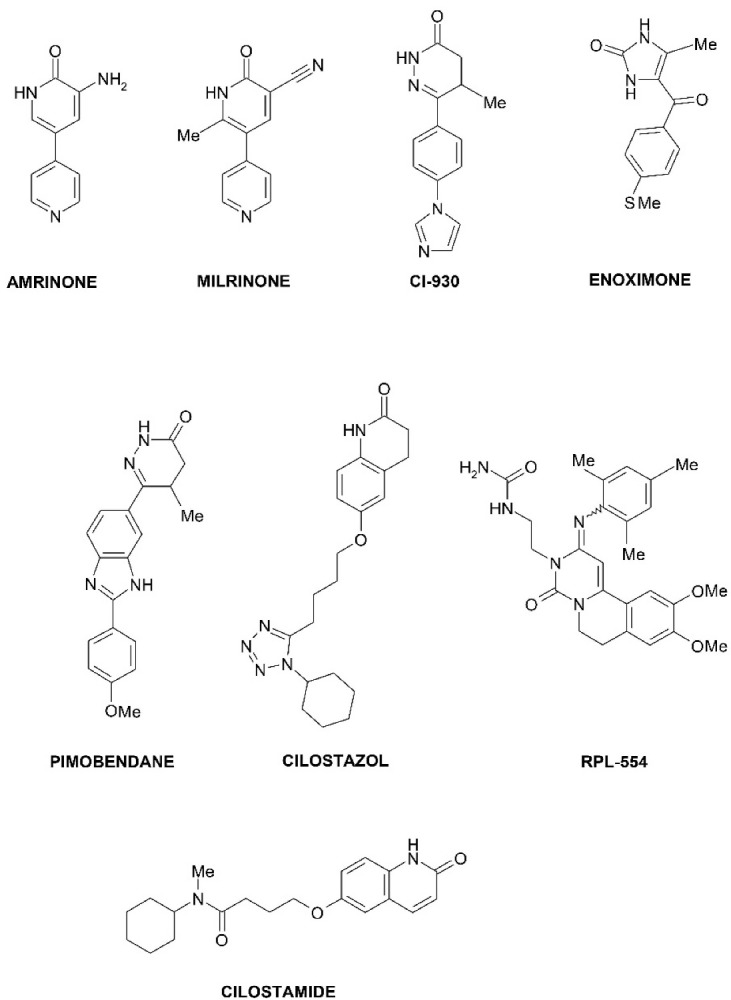
The chemical structures of known selective PDE3 inhibitors.

 PDE3 inhibitors, as pharmaceuticals, have been developed for the treatment of acute heart failure, cardiogenic shock, and as vasodilators (Figure 2). Examples of PDE3 inhibitors used in medicine are amrinone (a drug for the treatment of heart failure by increasing myocardial contractions due to increased concentrations of Ca^2+^ ions),^1^ milrinone (pulmonary vasodilator used in heart failure),^19^ enoximone (used in the treatment of congestive heart failure),^20^ pimobendan (a veterinary drug used to treat heart failure in animals),^21^ cilostazol (used in peripheral vascular disease and stroke prevention),^22^ cilostamide (highly selective PDE3 inhibitor). Cilostamide and its derivatives are widely used in studies as highly selective PDE3 inhibitors in combination with other drugs to obtain positive inotropic, chronotropic, antiarrhythmic effects.^23^

###  New prospective PDE3 inhibitors based on phosphorylated oxazole derivatives

 According to the literature, the number of oxazole derivatives with pronounced pharmacological activity, which has found practical application in medicine, is limited.^24-26^ A new series of original compounds of phosphorylated 1,3-oxazole derivatives (laboratory code ОVP-1, ОVP-2, ОVP-3, ОVP-4, ОVP-5, ОVP-6, ОVP-7, ОVP-8, ОVP-9, OVP-10) was synthesized in the Department of Bioactive Nitrogen-Containing Heterocyclic Bases Chemistry of the Institute of Bioorganic Chemistry and Petrochemistry (Kyiv, Ukraine). In screening experiments on isolated segments of the descending part of the thoracic rats’ aorta of phosphorylated oxazole derivatives OVP-1-OVP-10, the maximal vasodilating effect was detected in compound OVP-1 (full chemical name – diethyl ester of 5-N-alkylamino-2- N-benzoyl- (4-methylbenzylidene) glycyl] amino methyl} -1,3-oxazol-4-ylphosphonic acid), which in concentration of 10^-5^ M reduced the amplitude of the contraction force initiated by adrenaline 30.9%, (*P* < 0.05) relative to baseline.^10^ It is proved that the leader compound OVP-1 belongs to the V class of toxicity «practically non-toxic compounds» at intraperitoneal administration and to the VI class of toxicity «relatively harmless compounds» at intravenous administration. The presence of antihypertensive action of OVP-1 on models of acute and sustained arterial hypertension has been experimentally proved.^11^ Because the antihypertensive activity of OVP-1 may be related to its effect on the synthesis of vasodilating factor cGMP, the effect of the oxazole derivative on the change in the activity of its regulator, the PDE enzyme, was investigated.

 The docking method was used to further investigate the potential molecular mechanisms of the antihypertensive action of phosphorylated oxazole derivatives.

## Materials and Methods

 An experimental study of phosphorylated oxazole derivatives effect on phosphodiesterase activitywas performed on white mature Wistar rats of both sexes. Sustained hypertension was modeled by salt loading − salt drinking (1% sodium chloride solution) with free access to it for 21 days.^27^ To study the activity of PDE took blood serum, heart and aorta on the 22nd day after the start of modeling of stable hypertension. Determination of PDE activity was performed by fluorimetric method using umbelliferon.^28^ Aliquots of samples (50 μL) were added to the incubation medium of the following composition: 5 mmol MgCl_2_, 40 mmol NaCl, 50 μL cAMP (final concentration 1.9 mm) and 20 μL umbelliferon (final concentration 10^-6^ M). Next, the samples were incubated for 20 minutes at a temperature of 20°C. The RF-510 fluorometer (Shimadzu, Japan) was then measured for fluorescence intensity at an excitation wavelength of 350 nm and a maximum fluorescence wavelength of 442 nm. PDE activity was expressed in conventional units per minute and per 1 mg of protein.

###  Investigation of molecular mechanisms of phosphorylated oxazole derivatives action on PDE

 The AutoDockTools (ADT) program (ver. 1.5.6)^29^ was used due to its wide possibilities in studying the structure of active protein centers, studying the processes of binding of ligands to proteins, appropriate assessment of structural and chemical complementarity of proteins with ligands and obtaining energy complexation parameters. The ADT program was used to obtain a docking-compatible format of protein and ligand structure and to create a grid box. The crystal structure of the catalytic domain of H. sapiens PDBID: 1SO2 phosphodiesterase 3B catalyst in combination with a Merck1 inhibitor was used as the starting protein structure. For molecular docking, the APDE3 subunit was used, which was isolated from the overall structure of the enzyme and stored as a pdb file using Accelrys DS (ver. 2.5.5).^30-32^

 In the process of preparation of the macromolecule − A-subunit of PDE3 in the ADT program used the method «noBondOrder». Hydrogen atoms for all atomic groups were added to the structure of the macromolecule. All atoms of the macromolecule were renumbered. The PDE3 macromolecule with the calculated and added partial charges by the Gasteiger method was saved in the PDBQT format.

 The ligand structures of OVP-1, OVP-6, OVP-7 and OVP-10 (selected for comparative docking analysis) were constructed and saved in Mol2 format using ChemAxon Marvin Sketch. Ligand optimization and energy minimization were performed by Avogadro v1.1.1 using the «Autooptimization tool» using the MMFF94s force field and the «steepestdescent» algorithm. The partial charges and torsion angles of the ligands were changed using ADT and saved in PDBQT format.^19^

 Thus, the prepared protein structure of the optimized ligands was used as input files for molecular docking using AutoDock Vina 1.1.2.^29^ The AutoGrid subprogram was used to prepare the grid map and create the grid box. The center of the MERCK 1 ligand with coordinates x = 39.593, y = 67.686, z = 85.156 was calculated as the center of boxing. A grid of 30-30-30 points with a grid spacing of 1.0 Å was formed around this center. Molecular docking of one ligand into the active site occurred within 10 minutes. All docking procedures were performed on a computer running Windows 10 LTSC 1809 with AMDRyzen 7 2700X (3.70 GHz) processor and 8 GB of RAM. The Accelrys DS software package was used to visualize and study ligand-protein interactions.

## Results and Discussion

###  The experimental study of phosphorylated oxazole derivatives effect on phosphodiesterase activity

 One of the main mechanisms of the signaling action of NO is a direct effect on soluble guanylate cyclase in vascular smooth muscle tissue, which stimulates the synthesis of cGMP. The last one which can reduce the sensitivity of myofilaments to Ca^2+^ ions and cause vasodilation. Regulation of the effect of cGMP is partially provided by cellular PDE. The PDE3 family displays dual specificity with high affinity for both cAMP and cGMP. Furthermore, PDE3 shows a relatively high affinity for cGMP, which acts as a competitive inhibitor of cAMP hydrolysis, creating the so-called positive cGMP-to-cAMP crosstalk. In the cardiovascular system, mainly in the vascular SM, PDE3A is the predominant isoform found, while PDE3B is more highly expressed in cells involved in the regulation of glucose and lipid metabolism. Moreover, a mutated PDE3A gene drives mechanisms increasing the peripheral vascular resistance which may lead to hypertension.^16^ With the development of hypertension in conditions of salt load in the serum increases the content of aldosterone, accompanied by an increase in blood pressure.^33^ Therefore, interest in the activity of PDE in studies on the model of salt hypertension is also due to the participation of this enzyme in the regulation of aldosterone secretion in the glomerular zone of the kidneys against the background of reduced intracellular cAMP.^11^

 The results of the experiment on the effect of OVP-1 as a leader compound of phosphorylated oxazole derivatives^28^ on the total activity of PDE in the serum, heart and aorta of rats with hypertension are shown in Table 1. In rats with hypertension, PDE activity was increased by 73.4% (45.54 ± 2.36 CU, *P* < 0.05) in the aorta, 61.1% (14.16 ± 0.64 CU, *P*< 0.05) in the heart and 34.8% (8.64 ± 0.35 CU, *P* < 0.05) in the serum compared to intact animals.

**Table 1:** The effect of OVP-1 on the activity of PDE in the aorta, heart and serum of rats with sustained hypertension, CU (n = 12)

**Groups of animals**	**PDE activity**		
	**Aorta**	**Heart**	**Blood serum**
Intact (normotensive)	26.27 ± 1.44	8.79 ± 0.37	6.41 ± 0.28
Hypertension (control pathology)	45.54 ± 2.36*	14.16 ± 0.64*	8.64 ± 0.35*
% Changes	+ 73.4^a^	+ 61.1^a^	+ 34.8^a^
Hypertension + OVP-1 12.5 mg/kg	40.82 ± 2.89*	12.34 ± 0.42*^#^	7.95 ± 0.21*
% Changes	+ 55.4^a^ -10.4^b^	+ 40.4^a^ -12.9^b^	+ 24.0^a^ -8.0^b^
Hypertension + OVP-1 25 mg/kg	34.37 ± 1.83*^#^	10.94 ± 0.52*^#^	7.38 ± 0.37*^#^
% Changes	+ 30.8^a^ -24.5^b^	+ 24.5^a^ -22.7^b^	+ 15.1^a^ -14.6^b^
Hypertension + OVP-1 50 mg/kg	28.86 ± 1.87^#^	9.12 ± 0.19^#^	6.91 ± 0.21^#^
% Changes	+ 9.9^a^ -36.6^b^	+ 3.8^a^ -35.6^b^	+ 7.8^a^ -20.0^b^
Hypertension + amlodipine 1.5 mg/kg	38.65 ± 2.96*	11.55 ± 0.24*^#^	7.13 ± 0.17*^#^
% Changes	+ 47.1^a^ -15.1^b^	+ 31.4^a^ -18.4^b^	+ 11.2^a^ -17.5^b^
Hypertension + nebivolol 10 mg/kg	34.67 ± 3.00*^#^	9.07 ± 0.20^#^	6.86 ± 0.17^#^
% Changes	+ 32.0^a^ -23.9^b^	+ 3.2^a^ -35.9^b^	+ 7.0^a^ -20.6^b^

Notes: ^a^ % changes relative to the intact group; ^b^ % changes in the group of control pathology; * reliability compared to the intact group, *Р* < 0,05; ^#^ reliability compared with the control pathology group, *Р* < 0.05; n ˗ number of animals in the group.

 In the group of animals treated with OVP-1 at a dose of 12.5 mg/kg (1/2 ED_50_), there was a significant decrease in the activity of PDE in the heart by 12.9% (12.34 ± 0.42 CU, *P* < 0.05) relative to the group with control pathology, and in the aorta and serum there was a decrease in the form of a trend of 10.4% (40.82 ± 2.89 CU, *P* > 0.05) and 8.0% (7.95 ± 0.21 CU, *P* > 0.05). Under the influence of OVP-1 at a dose of 25 mg/kg (ED_50_) was observed a decrease in the studied parameter in the aorta by 24.5% (34.37 ± 1.83 CU, *P* < 0.05), in the heart - by 22.7% (10.94 ± 0.52 CU, *P* < 0.05), in blood serum - by 14.6% (7.38 ± 0.37 CU, *P* < 0.05 ) compared with a group of rats with hypertension. The introduction of OVP-1 at a dose of 50 mg/kg (2ED_50_) led to the restoration of PDE activity almost to the values observed in the intact group (in the aorta - 28.86 ± 1.87 CU, in the heart - 9.12 ± 0.19 CU, in blood serum – 6.91 ± 0.21 CU). A similar effect in the heart and serum was studied under the influence of nebivolol and amounted to 9.07 ± 0.20 and 6.86 ± 0.17 CU, respectively. However, the effectiveness of nebivolol in the aorta was lower compared to OVP-1 50 mg/kg and was 34.67 ± 3.00 CU. In turn, amlodipine significantly reduced the activity of PDE in the heart by 18.4% (11.55 ± 0.24 um., *P* < 0.05) and in serum - by 17.5% (7.13 ± 0.17 um., *P* < 0,05), while in the aorta this effect was observed as a trend relative to the group with control pathology. The established data may indicate the possibility of implementing the vasodilating effect of OVP-1 by influencing the latter to increase the synthesis of cGMP due to inhibition of PDE activity.

 The obtained experimental results showed the presence of the compound OVP-1 vasodilating effect and antihypertensive effect associated with PDE3.^11,28^

 Reduction in PDE3A expression has been reported in human failing hearts.Angiotensin II and isoproterenol induce sustained downregulation of PDE3A expression and upregulation of inducible cAMP early repressor (ICER) expression in cardiac myocytes.CER induction represses PDE3A expression, leading to activation of cAMP/PKA signaling, which contributes to ICER expression. PDE3B expression requires peroxisome proliferator–activated receptor γ (PPARγ) and is upregulated during differentiation to adipocytes in 3T3-L1 cells. In contrast, treatment of fully differentiated 3T3-L1 adipocytes with TNFα reduces PDE3B expression. In addition, C_2_-ceramide (a short-chain ceramide analog), which is involved in TNFα signaling, decreases the level of PDE3B protein. Troglitazone, an antidiabetic drug, antagonizes ceramide action on PDE3B expression. On the other hand. PKA-mediated PDE3A activation functions as negative-feedback regulation in cAMP signaling, and PKB-mediated PDE3B activation in adipocytes greatly contributes to insulin action. Little is known about PKA- and PKB-mediated phosphorylation of rat PDE3A, which has potential phosphorylation sites. With regard to PDE3B, human PDE3B contains typical PKB (Ser295) and PKA phosphorylation motifs. However, there is no report on the phosphorylation of these sites.^34^ PDE3-selective inhibitors such as amrinone, enoximone, and milrinone have been used clinically to acutely treat congestive heart failure. In human myocardium, PDE3 inhibitors increase the rate and magnitude of developed force as well as enhance the rate of muscle relaxation and have antihypertensive effect. Concurrently, in human vasculature, PDE3 inhibition reduces total peripheral and pulmonary vascular resistance and enhances coronary blood flow.^35^ Both milrinone and enoximone have been shown to reduce systemic, pulmonary, and coronary vascular resistance when administered in inotropic doses.^36^ Thus PDE3 inhibitors are powerful drugs for the acute treatment of the congestive heart failure because of simultaneous increased contractility of the heart and decreased resistance of blood flow through the vasculature. However, based on results of experimental studies can be considered as a new platform for further studies as phosphodiesterase III inhibitors with antihypertensive activity.

 Therefore, after proving the effectiveness of phosphorylated oxazole derivatives to reduce the activity of phosphodiesterase on the experimental model of hypertension, our next step was to determine the molecular mechanisms of this effect.

###  Calculation of structural conformation and energy minimization

**Figure 3:**
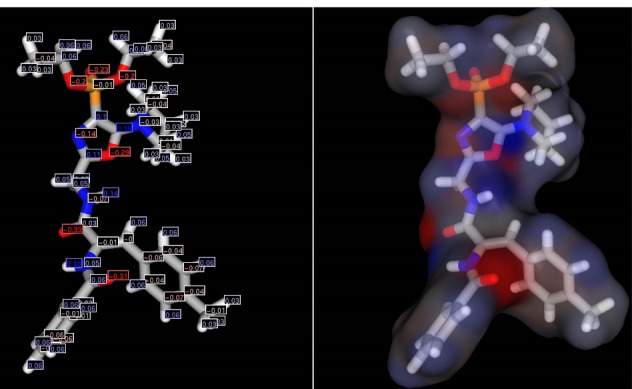
Conformation of the molecule OVP-1 with the calculated partial charges.

 Partial charges were calculated for the obtained structural conformation using the ChemAxon MarvinSketch program (Figure 3).

 Negative charges are calculated for oxygen atoms of the phosphonate group in the range from -0.2 to -0.23, for nitrogen atoms (-0.14) and oxygen (-0.29) of the oxazole cycle, and positive partial charges - on carbon atoms (0.1-0.18). The calculated negative oxygen charges and positive for hydrogen glycyl chain ranged from -0.31 to -0.33 and from 0.14 to 0.16, respectively. The presence of such an arrangement of positive and negative charges is likely to determine the reactivity of the compound and the possibility of interactions with amino acid residues in the active site of the enzyme.

###  Molecular docking of ligands of phosphorylated oxazole derivatives to PDE3 active site

**Figure 4:**
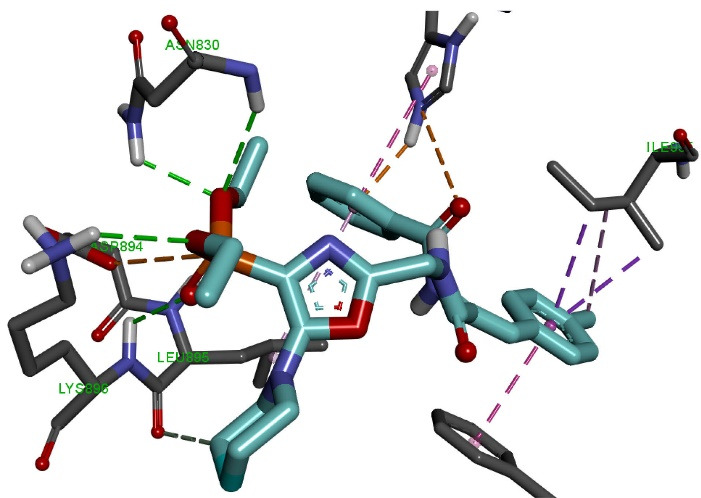
Molecular docking of OVP-1 to the active site of PDE3.

**Figure 5:**
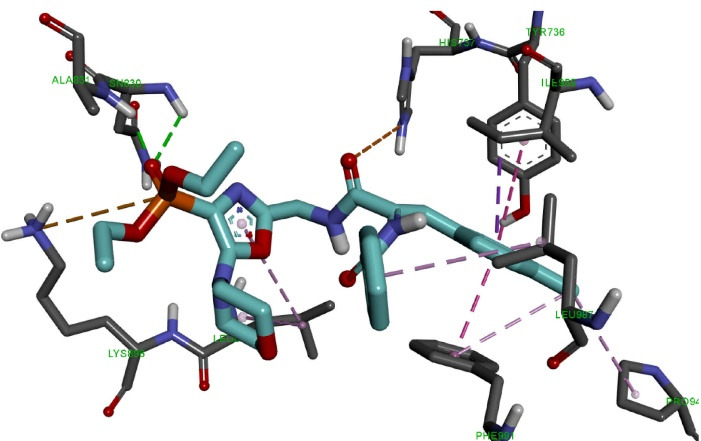
Molecular docking of OVP-6 to the active site of PDE3.

**Figure 6:**
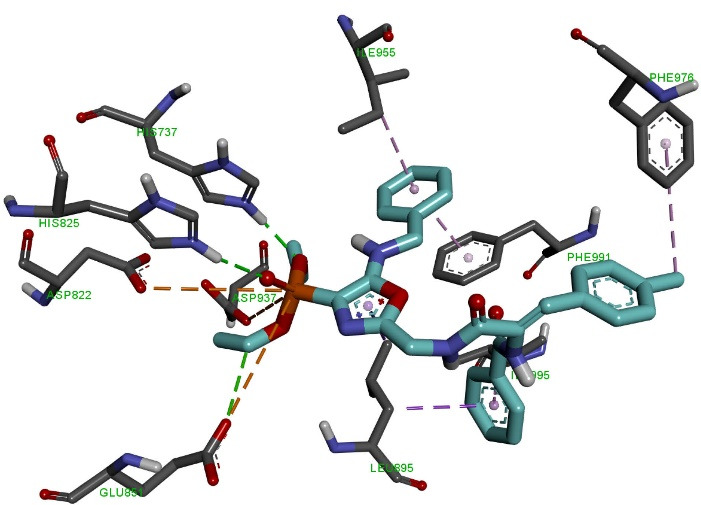
Molecular docking of OVP-7 to the active site of PDE3.

**Figure 7:**
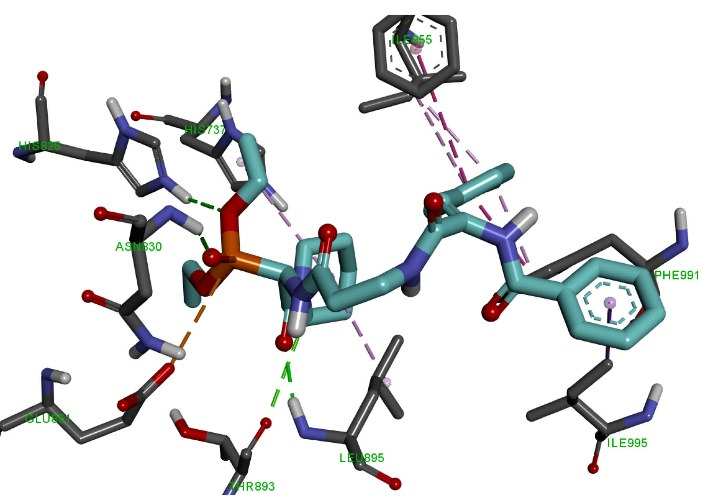
Molecular docking of OVP-10 to the active site of PDE3.

 Molecular docking of the most active compounds OVP-1, OVP-6, OVP-7, OVP-10 to the active center of PDE3 is presented in Figures 4-7.

 Docking validation was performed by re-docking the Merck1 ligand to the active PDE3 site. The obtained ligand-protein complex showed ΔG = –10.1 kcal/mol. The RMSD value for all ligand atoms did not exceed 2 Ǻ. Molecular docking of cilostamide as a known PDE3 inhibitor was also performed (Table 1).

 Molecular docking of the OVP-1 ligand (Figure 4) to the PDE3 active site showed that the phosphonate group forms four hydrogen bonds of length 2.42-3.04 Å with amino acid residues Asn830, Lys896 and electrostatic interaction (4.06 Å) with Asp894. The piperidine ring forms one hydrogen bond (3.52 Ǻ) with the amino acid Leu895. The side methylphenyl ring of the compound forms four hydrophobic bonds with a length of 3.83-4.66 Ǻ with Ile955 and Phe991.

 The final phenyl ring and the nearest oxygen atom form two electrostatic interactions (2.69 Ǻ, 4.32 Ǻ) with His737, as well as two hydrophobic interactions (4.67 Ǻ, 4.99 Ǻ) with His737 and Leu895.

 Molecular docking of the OVP-6 ligand (Figure 5) to the PDE3 active site showed that the phosphonate group formed two 1.95-2.73 Å hydrogen bonds with amino acid residues Asn830, Ala831, and an electrostatic interaction (5.12 Å) with Lys896. The piperidine and oxazole rings form one hydrophobic interaction (5.24-5.36 Ǻ) with the amino acid Leu895. The side methylphenyl ring of the compound forms five hydrophobic interactions of length 3.95-5.85 Ǻ with Ile955, Tyr736, Pro941 and Phe991. The final phenyl ring and the nearest oxygen atom form electrostatic and hydrophobic interactions (5.25 Ǻ, 5.29 Ǻ) with His737.

 Molecular docking of the OVP-7 ligand (Figure 6) to the active PDE3 center showed that the phosphonate group forms three hydrogen bonds with a length of 2.09-3.36 Å with amino acid residues His737, His825, Glu851 and two electrostatic interactions (5.41 Å, 5.52 Ǻ) with Asp822 and Glu851. The oxazole ring forms one hydrophobic interaction 3.93Ǻ with the amino acid Leu895. The first phenolic ring forms two hydrophobic interactions (3.52 Ǻ, 4.06 Ǻ) with the amino acids Ile955 and Phe991. The side methylphenyl ring of the compound forms a 5.06 Ǻ hydrophobic bond with Phe976. The final phenyl ring forms two hydrophobic interactions (3.97 Ǻ, 4.50 Ǻ) with Leu995 and Leu895.

 Molecular docking of the OVP-10 ligand (Figure 7) to the PDE3 active site showed that the phosphonate group forms two hydrogen bonds of length 2.09-3.36 Å with amino acid residues His825, Asn830 and electrostatic interaction (4.13 Å) with Glu851. The piperidine ring and the oxygen atom form two hydrogen bonds of length 2.00-3.77Å with amino acid residues Leu895, Thr893, as well as two hydrophobic interactions (4.61 Ǻ, 5.22 Ǻ) withLeu895 and His737. The side methylphenyl ring of the compound forms five hydrophobic interactions with a length of 3.87-5.09 а with the amino acids Phe991, Ile955 and His737. The final phenyl ring forms a 3.40 hydrophobic interaction with Ile995.

**Figure 8:**
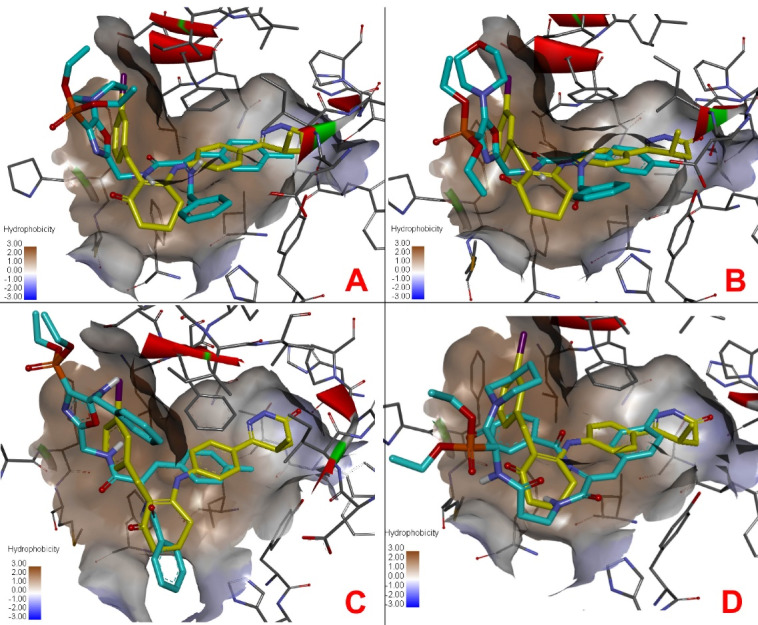
Docking positions of test compounds (blue) compared to Merck1 inhibitor (yellow) in the active site of PDE3. (A) compound OVP-1, (B) compound OVP-6, (C) compound OVP-7, (D) compound OVP-10.

 The docking positions of the compounds (Figure 8) showed some similarity in the active site compared to the Merck1 inhibitor. A comparative docking of a known cilostamide inhibitor to the active site of PDE3 was also performed. The results of molecular docking of the test compounds are presented in Table 2.

**Table 2:** The results of molecular docking of phosphorylated oxazole derivatives ligands to PDE3 active site

**Compound**	**∆G, kcal/mol**	**Number of hydrogen bonds and** **electrostatic interactions**
Merck1	– 10.1	6
Cilostamide	– 9.1	5
OVP-1	– 9.1	8
OVP-6	– 8.7	4
OVP-7	– 8.9	5
OVP-10	– 9.0	5

 The calculated results of molecular docking of ligands OVPs to the active site of PDE3 (Table 2) showed that all test compounds have a common type of complexation due to phosphonate groups, piperidine rings, side and terminal phenyl and methylphenyl groups. The binding energy (ΔG) of the formed ligand-protein complexes for compounds OVP-1, OVP-6, OVP-7, OVP-10, Merck1 and cilostamide was -9.1, -8.7, -8.9, -9.0, -10.1 and - 9.1 kcal / mol, respectively. Amino acid residues Asn830, Lys896, Asp894, Leu895, Ile955, Phe991, His737, Ala831, Tyr736, Pro941, His825, Glu851, Asp822, Phe976 and Thr893 play a key role. In addition, this energy of the formed ligand-protein complexes of the test compounds correlates with their experimental activity, which allows us to consider them as potential PDE3 inhibitors with antihypertensive action.

## Conclusion

 Due to the possibility of phosphorylated oxazole derivatives antihypertensive action due to the influence on the synthesis of vasodilating factor cGMP, which is also affected by the signaling effect of NO, the change in the activity of its regulator − PDE under the action of oxazole derivative was observed. This may indicate the possibility of the development of vasodilating action of OVPs by the influence of the latter on the increase in cGMP synthesis due to inhibition of PDE activity. The calculated results of molecular docking of ligands OVPs to the active site of PDE3 showed that all test compounds have a common type of complexation due to phosphonate groups, piperidine rings, side and terminal phenyl and methylphenyl groups. Thus, the analysis of the obtained results both *in vivo* and *in silico* showed that phosphorylated oxazole derivatives represent a new platform for further studies as phosphodiesterase III inhibitors with antihypertensive activity.

## Acknowledgments

 The authors are grateful to the management and the head of the Department of Bioactive Nitrogen-Containing Heterocyclic Bases Chemistry of the Institute of Bioorganic Chemistry and Petrochemistry to support the research work by synthesis of phosphorylated oxazole derivatives.

## Competing Interests

 The authors declare no conflict of interest.

## Ethical Approval

 Not applicable.
